# Oncoplastic breast surgery to treat recurrent granulomatous abscess: A new approach

**DOI:** 10.1016/j.ijscr.2021.106440

**Published:** 2021-09-23

**Authors:** Mohamed Aymen Ferjaoui, Ramzi Arfaoui, Slim Khedhri, Henda Mustapha, Monia Malek, Khaled Neji

**Affiliations:** a Department B of Gynecologic Surgery, Tunis Maternity Center, Tunis Medical School, El Manar University, Tunisia; b Maternity Department, Tunis Military Hospital, Tunis Medical School, El Manar University, Tunisia; c Unit of Reproductive Medicine, Farhat Hached Hospital, Sousse Medical School, University of Sousse, Tunisia

**Keywords:** Granulomatous mastitis, Breast surgery, Oncoplasty, Case report

## Abstract

**Introduction and importance:**

Granulomatous mastitis is a rare chronic and benign inflammatory breast disease with challenging diagnosis and management. No commonly recognized recommendations are established. Despite of medical and surgical approaches, relapse rate remains high.

**Case presentation:**

A 43-year-old patient with a history of granulomatous mastitis presented recurrent breast abscess associated with skin fistula. She underwent Racquet mammoplasty procedure inspired from oncoplastic techniques. Medical and cosmetic outcomes were satisfactory.

**Clinical discussion:**

Granulomatous mastitis is challenging to diagnose and can be confused with inflammatory breast malignancies. It's associated with high relapse rate. Management of granulomatous mastitis is complex. Its treatment varies from medical management based on steroid therapy and immunosuppressants to surgical approach. In case of recurrent breast abscess, surgical techniques inspired from oncoplastic breast surgery, can be used to improve cosmetic outcome.

**Conclusion:**

Oncoplastic surgical technic may be considered as an efficient procedure to manage recurrent breast granulomatous abscess.

## Highlights

• Granulomatous mastitis is a rare challenging condition.
• Up to now, there isn’t a commonly recommended therapeutic strategy.
• Relapse rate is high with an unsatisfactory cosmetic outcome.
• Oncoplastic surgery may provide cosmetic result with low relapse rate.

## 1 Introduction

Granulomatous mastitis is a rare benign inflammatory breast disease. This is a chronic condition with unknown etiology affecting usually young women. Patients with granulomatous mastitis can present painful breast lump, skin inflammation and erythema or chronic breast abscess [1], [2]. The diagnosis is challenging due to clinical presentation similarity with breast cancer. In fact, it can be confused with inflammatory breast malignancy [3].

Management of granulomatous mastitis is laborious and complex. Up to now, no validated universal recommendation was reported. The treatment varies from medical management based on steroid therapy and immunosuppressants to surgical approach including simple or wide excision and mastectomy for recurrent abscess [4]. Medical and surgical approaches may be associated to improve results.

Aesthetic and cosmetic outcome after surgical excision remains poor and unsatisfactory because of important local inflammatory reaction and wide tissue resection. It impacts negatively on patient's self-imaging and leads to psychologic disorders.

To improve cosmetic outcome, surgical techniques inspired from oncoplastic breast surgery, can be used to manage recurrent breast abscess [5], [6].

We report a case of recurrent breast abscess with a skin fistula in a patient with history of granulomatous mastitis. Racquet mammoplasty technique was performed.

This case report aims to explain how to perform Racquet mammoplasty surgical technique and to assess its usefulness to decrease abscess relapse rate and to preserve cosmetic outcome.

The work has been reported in line with the SCARE criteria [7].

## 2 Case report

A 43-year-old patient with three-year-history of granulomatous mastitis, was initially treated by six-month concomitant steroid and antibiotic therapy without any local improvement. No comorbidities were reported. She presented a recurrent breast abscess associated with skin fistula located in the upper outer quadrant of left breast. Breast ultrasound showed multiple contiguous hypoechoic masses with posterior enhancement associated to fluid collection communicating with skin fistula. Breast cancer diagnosis was rejected after needle core biopsy and histologic examination.

We decided to perform wide excision to remove diseased area and skin fistula. Surgery aims to ovoid recurrence and to preserve aesthetic outcome.

Our surgical strategy was inspired from oncoplastic breast approaches used to treat tumors in the upper outer quadrants. Racquet mammoplasty was performed.

### 2.1 The surgical technique

**Fig. 1:**
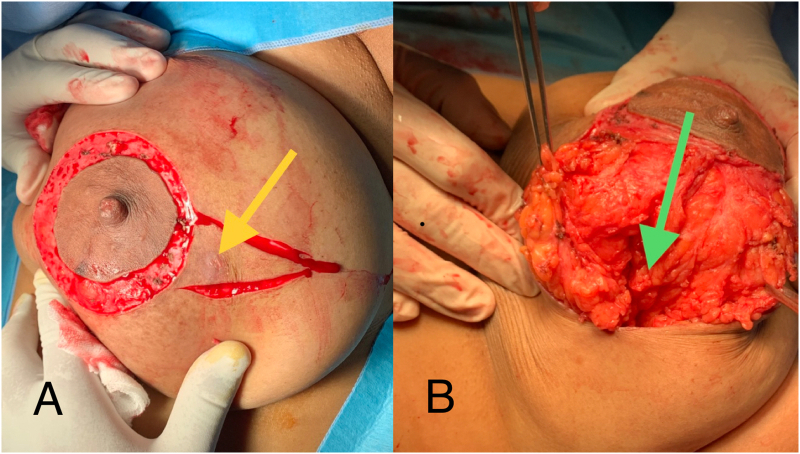
A: A round Block associated with a large outer incision surrounding skin fistula (yellow arrow). B: Breast defect (green arrow) is covered by dissected upper and lower glands. (For interpretation of the references to colour in this figure legend, the reader is referred to the web version of this article.)

Under general anesthesia, the patient was placed on supine position. Round bloc incision was performed to recentralize the NAC (nipple areola complex) position after abscess excision. It was associated with an outer large racquet incision surrounding the skin fistula. A large portion of upper outer quadrant was excised. Upper and lower gland were dissected and mobilized to cover the residual cavity using layered suture (Fig. 1).

Retro areolar gland was dissected and separated from the NAC to provide maximal mobility of central gland for volume redistribution and breast reshipment.

**Fig. 2:**
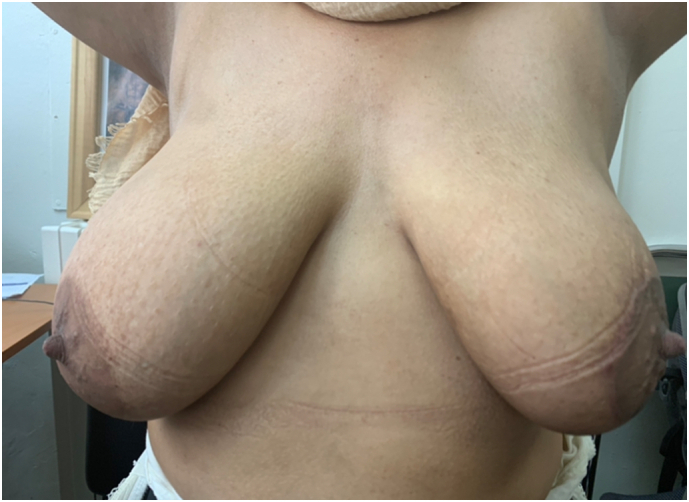
Six-month post-operative result.

**Fig. 3:**
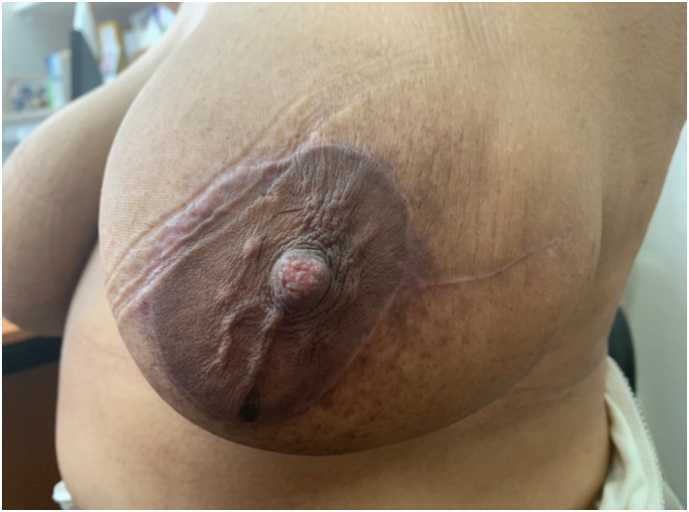
Peri areolar and radial scars.

After covering the breast defect, the NAC was replaced to have breast harmonious and symmetric aspect (Fig. 2). After surgery, the patient has an external radial and *peri* areolar scars (Fig. 3). Post-operative period was uneventful and the surgery was uncomplicated.

Six months follow-up showed no recurrence with good cosmetic outcome.

## 3 Discussion

Granulomatous mastitis is a rare idiopathic chronic breast inflammatory disease. It affects usually young women in childbearing age [1], [8]. Clinical presentations vary from breast painful lump to recurrent abscess mimicking breast carcinoma and tuberculosis mastitis [2]. The diagnosis is based on histologic examination which aims to exclude malignancy and to confirm granulomatous mastitis [3], [8], [9]. Up to now, there is no commonly recognized management. The most established practice indicates steroid and antibiotic therapies associated with surgical excision [10]. High recurrence rate was reported in almost all series, it can reach 50% [11].

The management of relapsed abscess in case of granulomatous mastitis is challenging. Most authors indicate wild surgical excision [3]. In fact, simple excision is complicated by skin retraction, unsightly scars and breast deformity impacting negatively on patient's self-imaging and psychology.

Many surgical techniques are described to improve aesthetic and cosmetic outcome. They are inspired from oncoplastic surgical procedures indicated in breast cancer conservative management [5], [6], [12].

In our case, we present an oncoplastic procedure performed to treat relapsed granulomatous abscess: Racquet mammoplasty technic.

Firstly, oncoplastic breast surgery was indicated to improve cosmetic outcome in breast cancer surgery. Its indication was enlarged to manage benign tumors [13]. Actually, those techniques are recommended in palliative setting to remove advanced and recurrent breast cancer providing good psychological outcome and comfort for patients with relatively short life expectancy [14].

Oncoplastic procedures performed in recurrent complex suppurative and inflammatory diseases aren't well documented and few series are reported. The largest one was the study published by Giacalone et al. They performed round bloc oncoplastic technique for recurrent retro areolar abscess, it was an efficient and feasible procedure to excise breast abscess and to ovoid relapses [5].

Because of lack of literature reviews and reports concerning breast oncoplastic surgery in relapsed abscess, randomized trials are needed to evaluate this technique and to assess its usefulness in recurrent breast granulomatous abscess.

## 4 Conclusion

Granulomatous mastitis complicated by recurrent abscess is a rare and challenging condition requiring surgical excision. Oncoplastic breast procedures may be an efficient and feasible technique providing good medical and cosmetic outcome for well selected patients.

## Provenance and peer review

Not commissioned, externally peer-reviewed.

## Funding

No source of funding to declare.

## Ethical approval

This case report was written with the agreement of ethical committee of Tunis maternity center.

## Consent

Written informed consent was obtained from the patient for publication of this case report and accompanying images. A copy of the written consent is available for review by the Editor-in-Chief of this journal on request.

## Author contribution

**Mohamed Aymen Ferjaoui and Ramzi Arfaoui**: Surgeons.

**Slim Khedhri and Hinda Mustapha**: data collection and analysis.

**Monia Malek and Khaled Neji**: supervisors.

## Registration of research studies

This is not a “first in humans” report, so it is not in need of registration.

## Guarantor

Dr Mohamed Aymen Ferjaoui.

## Declaration of competing interest

No conflicts of interest to report.

## References

[1] Mohammed S., Statz A., Lacross J.S., Lassinger B.K., Contreras A., Gutierrez C. (2013). Granulomatous mastitis: a 10 year experience from a large inner city county hospital. J. Surg. Res..

[2] Kok K.Y., Telisinghe P.U. (2010). Granulomatous mastitis: presentation, treatment and outcome in 43 patients. The Surgeon.

[3] Chen L., Zhang X.Y., Wang Y.W., Zhao Q.F., Ding H.Y. (2019). Granulomatous lobular mastitis: a clinicopathological analysis of 300 cases. Zhonghua Bing Li Xue Za Zhi.

[4] Poovamma C.U., Pais V.A., Dolas S.C., Prema M., Khandelwal R., Nisheena R. (2014). Idiopathic granulomatous mastitis: a rare entity with a variable presentation. Breast Dis..

[5] Giacalone P.L., Rathat G., Fournet S., Rouleau C. (2010). Surgical treatment of recurring subareolar abscess using oncoplastic techniques. J. Visc. Surg..

[6] Bognar G., Ledniczky G., Lóderer Z. (2013). Aesthetic outcome as a goal using pectoral muscle-strip in recurrent subareolar abscess of the breast and for double subdermal flap in modelling of the inverted nipple. Arch. Gynecol. Obstet..

[7] Agha R.A., Franchi T., Sohrabi C., Mathew G., Kerwan A. (2020). The SCARE 2020 guideline: updating consensus surgical CAse REport (SCARE) guidelines. Int. J. Surg..

[8] Hello M., Néel A., Graveleau J., Masseau A., Agard C., Caillon J. (2013). Idiopathic granulomatous mastitis. Rev. Med. Interne.

[9] Wolfrum A., Kümmel S., Theuerkauf I., Pelz E., Reinisch M. (2018). Granulomatous mastitis: a therapeutic and diagnostic challenge. Breast Care (Basel).

[10] Skandarajah A., Marley L. (2015). Idiopathic granulomatous mastitis: a medical or surgical disease of the breast?. ANZ J. Surg..

[11] Ocal K., Dag A., Turkmenoglu O., Kara T., Seyit H., Konca K. (2010). Granulomatous mastitis: clinical, pathological features, and management. Breast J..

[12] Yukawa M., Watatani M., Isono S., Fujiwara Y., Tsujie M., Kitani K. (2015). Management of granulomatous mastitis: a series of 13 patients who were evaluated for treatment without corticosteroids. Int. Surg..

[13] Ren J., Jin L., Leng B., Hu R., Jiang G. (2018). Surgical excision and oncoplastic breast surgery in 32 patients with benign phyllodes tumors. World J. Surg. Oncol..

[14] Veronesi G., Scanagatta P., Goldhirsch A., Rietjens M., Colleoni M., Pelosi G. (2007). Results of chest wall resection for recurrent or locally advanced breast malignancies. Breast (Edinburgh, Scotland).

